# Congratulations on the 60th anniversary of the Japanese Society of Nuclear Medicine

**DOI:** 10.1007/s12149-024-02008-1

**Published:** 2025-02-01

**Authors:** Seigo Kinuya

**Affiliations:** Kanazawa University Graduate School of Medical Science, Kanazawa, Ishikawa Japan

## Abstract

The Japanese Society of Nuclear Medicine (JSNM) celebrates its 60th anniversary this year. JSNM has been contributing a lot to the development of nuclear medicine, leading a number of international activities. New procedures both in diagnosis and treatment have been continuously developed. JSNM will work hard for the better management of patients.

On behalf of the members of the Japanese Society of Nuclear Medicine (JSNM), I would like to congratulate its 60th anniversary. I would appreciate all people in this field for supporting society activities for a long time. The Japan Radioisotope Association (JRIA) is essential for JSNM on the delivery and release of radioactive materials and the implementation of training courses for therapeutic nuclear medicine. Administrative officers in governmental bodies have not hesitated to together discuss and adapt legislations to circumstances for new diagnostic and therapeutic maneuvers. My appreciation should also go to industrial people who continuously provide us with radiopharmaceuticals for our clinical activities. JSNM derived from the former organization of JRIA in 1961 as the research group on nuclear medicine and the first group meeting was held in this year. The group was reorganized in 1964, which led to the founding of JSNM. The society obtained the status of General Incorporated Association in 2008 after its recognition as a limited liability intermediate corporation in 2005. In 2017, then president of JSNM, Prof. Tomio Inoue of Yokohama City University, successfully held the congress of Asia Oceania Federation of Nuclear Medicine and Biology (AOFNMB) in Yokohama. Furthermore, JSNM hosted the 13th congress of the World Federation of Nuclear Medicine and Biology (WFNMB) in 2022 [1] nearly a half century after the 1st congress led by our predecessors of JSNM in 1974 [2]. I appreciated the leaders of JSNM, Prof. Inoue, Prof, Jun Hatazawa of Osaka University and the leaders of JSNM, who decided the initiation of promotive campaign for WFNMB in 2013. We, JSNM members, should be proud of such accomplishments and consider to continue these activities. International collaboration with societies in the world other than AOFNMB and WFNMB has been an important mission of JSNM. JSNM has worked together with the Society of Nuclear Medicine and Molecular Imaging (SNMMI), European Association of Nuclear Medicine (EANM) under the memorandum of understanding between them which enables designated sessions in each annual conference. Asian Regional Cooperative Council for Nuclear Medicine (ARCCNM), China-Japan-Korea (CJK) conference on nuclear medicine and others have been regularly held. Additionally, the Asia Nuclear Medicine Board has been established to certify nuclear medicine specialists. One more recent activity that should be emphasized is the Consortium of Universities and Institutions in Japan (CUIJ). This collaborative work with the International Atomic Energy Agency (IAEA) was first initiated at Osaka University under the initiative of Prof. Hatazawa in 2016, aiming to provide Asian young people with education and new information regarding nuclear medicine practices. A total of 11 Japanese institutions participated in 2018 and had been planning workshops or lecture courses. This kind of international work surely contributes to the development of nuclear medicine in this area. During these 60 years, a number of evolutions occurred in clinical practice and research works. Thanks to these advances, the management of patients has been continuously improving both in diagnosis and therapy. The efforts of all the people who contributed to the activities were surely respectful. Medical practices have always been changing. Management of patients used to be uniform in many disease entities, meaning that treatments were the same in patients with particular diagnosis. For instance, similar treatment regimens were adapted to patients suffering from particular malignancy. In such era, classical nuclear medicine techniques of images for the bone, liver, kidneys and so on could provide physicians with functional information of each organ. The scenario has been rapidly developing with genomic testing or molecular profiling where nuclear images have key roles that enables in vivo visualization of essential molecules of diseases. New radioligands have been also introduced as treatment options. The concept of radiotheranostics is currently essential in medical care. Nuclear medicine will take even a bigger role in medical practices and research works in the future. I am truly happy to have the opportunity to serve as the president of JSNM during the very last period of my academic carrier as a nuclear medicine physician. Practices that were thought to be dream stories in my early career are routinely performed in daily clinics now. Nuclear medicine will never end to acquire new ways for the wellness of people. I sincerely hope that the younger generation of JSNM members keep their will on further development of our field.
